# Development of a Community-Based Communication Intervention among Latin Caregivers of Patients Coping with Cancer

**DOI:** 10.3390/healthcare12080841

**Published:** 2024-04-16

**Authors:** Normarie Torres-Blasco, Lianel Rosario-Ramos, Carled Arguelles, Stephanie Torres Marrero, Tiffany Rivera, Zulay Vicente, Maria Elena Navedo, Rosael Burgos, Mayra Garriga, Maria del Carmen Pacheco, Betsy Lopez

**Affiliations:** 1 School of Behavioral and Brain Sciences, Ponce Health Sciences University, Ponce 00716, Puerto Rico; lrosario21@stu.psm.edu (L.R.-R.); carguelles23@stu.psm.edu (C.A.); storres21@stu.psm.edu (S.T.M.); tirivera21@stu.psm.edu (T.R.); zvicente21@stu.psm.edu (Z.V.); rburgos20@stu.psm.edu (R.B.); mgarriga20@stu.psm.edu (M.G.); mpacheco20@stu.psm.edu (M.d.C.P.); belopez20@stu.psm.edu (B.L.); 2 Ponce Research Institute, Ponce 00716, Puerto Rico; 3 Department of Medicine, University of Connecticut, Storrs, CT 06269, USA; menavedomd@gmail.com

**Keywords:** communication, cancer, dyads, community, providers, patients, caregivers, skills, strategies

## Abstract

**Background**: Cancer affects the emotional well-being of patients and caregivers, highlighting the need for effective communication strategies. This study explores a community-based communication intervention for Latino caregiver–patient dyads coping with cancer. The acceptability of the intervention, along with its associated facilitators and barriers, are crucial considerations. **Methods**: Three focus group interviews involved healthcare providers, community partners, patients, and caregivers to discuss the communication needs of this population and the components of a communication intervention while identifying facilitators and barriers to the intervention. Qualitative thematic content analysis was conducted using Nvivo v12, ensuring reliability through independent analysis and consensus building. **Results**: Participants (89% female, average age of 53) included patients (30%), caregivers (30%), community partners (25%), and healthcare providers (15%), and they discussed the overall acceptability of adapting a communication intervention, where they emphasized benefits for caregivers and patients, primarily through support groups. Communication strategies accepted by participants include psychological support, cancer education, assertive communication skills, and methods for improved interactions with healthcare providers and extended family. **Conclusions**: Participants’ responses align with the current literature, emphasizing problem-solving, mutual support, and communication strategies and underscoring the role of community partners. The study underlines the necessity for culturally tailored communication interventions for Latino families facing cancer.

## 1. Introduction

Cancer poses challenges that profoundly impact the physical, emotional, and psychological well-being of patients and their caregivers—comprehensive care identifies patient needs and preferences while supporting caregivers [1]. Caregivers play a critical role in the cancer care process, but their emotional well-being is closely tied to their ability to communicate openly with the patient [2]. Non-effective communication negatively impacts the caregiver’s quality of life [3] and can cause high levels of distress in the caregiver and patients [4]. Communication can hinder discussing fears and diagnosis concerns when discussing distressing topics and addressing complex issues [5]. Discussing fears and effective communication coping strategies alleviates some of this distress and can improve caregivers’ overall quality of life [6].

A meta-analysis has shown that culturally adapted treatments tailored for specific cultural groups are four times more effective than interventions provided to participants from various cultural backgrounds; those conducted in Latino participants’ native language (i.e., Spanish) are twice as effective as interventions conducted in English [7]. Though several psychotherapeutic interventions are designed for CST, none have been explicitly adapted for Latino patients and caregivers. Considering Latino communities’ unique cultural context and needs, exploring alternative avenues is imperative. Among Latinos, community partners and community intervention teams could effectively facilitate psychosocial intervention delivery [8]. These partners play a pivotal role in adapting interventions to align with the cultural and linguistic preferences of the Latino community [9]. The heterogeneity of Latino culture underscores the importance of recognizing and respecting the diversity of experiences, perspectives, and needs within Latino communities when designing interventions or providing services. Tailoring interventions to specific cultural contexts and engaging with community partners can help ensure that interventions are relevant, effective, and inclusive. Established community partners are trusted figures within the community, which helps build rapport and trust with patients, leading to better engagement and adherence to psychosocial interventions [10]. Among communities, support groups tailored for cancer patients offer numerous advantages, which include delivering emotional support and coping strategies to patients and caregivers coping with cancer [11]. These groups provide a safe and empathetic space where individuals facing cancer can share their experiences, seek guidance, and develop effective coping mechanisms [12]. Furthermore, it is essential to extend support to communities and provide training programs specifically designed to educate patients and family caregivers about the distinct needs and challenges faced by Latinos coping with cancer [13]. 

Effective communication interventions for Latinos coping with cancer are vital for addressing health disparities [14]. Given the complexity of addressing psychosocial needs (e.g., quality of life) among the patients and caregivers, a facilitator in this process could involve community partners and support groups to deliver effective psychosocial interventions for Latinos coping with cancer [8]. These community-based approaches can enhance access to culturally sensitive support services and improve overall well-being among Latino cancer patients and their families. This article describes an adaptation of a CST intervention and the associated acceptability, barriers, and facilitators to explore and adapt the CST among patients and caregivers coping with cancer. As Latino culture places a strong emphasis on social and community support, it is imperative to explore and adapt a CST to improve communication among patients and caregivers coping with cancer [15]. This communication intervention can facilitate the involvement of family and community members in the patient’s journey, which is crucial for coping with cancer [16].

## 2. Materials and Method

The methodology for this study is aimed at adapting a communication intervention within the targeted community. We guided our study with the Community Engagement Research (CER) framework [17,18]. The CER framework was used when the academic researchers (NTB) worked alongside community partners (R.B., M.G., M.P., B.L.) who (1) did not have formal academic research education, (2) were very familiar with and part of the patient and caregiver needs, and (3) were interested in having prior personal experience with the topic of the research (e.g., communication needs among patients and caregivers coping with cancer) [17,18]. The academic researcher (NTB) collaborated with four community partners (R.B., M.G., B.L., C.P.) who are community partners in rural towns in Southern Puerto Rico (Villalba, Lajas, Peñuelas, and Yauco), with personal experiences related to cancer. All community researchers obtained Community-Based Participatory Research training before participating. They selected the focus group methodology as it provides a suitable environment for understanding collective social action and accessing group beliefs about a psychosocial intervention [19]. 

Ethical approval for the study procedures was obtained from the Ponce Research Institute Institutional Review Board (IRB #2206107691A002), ensuring compliance with ethical standards. Recruitment of participants, including cancer patients, caregivers, and community partners, was facilitated by community partners (R.B., M.G., B.L., C.P.) from rural towns in Southern Puerto Rico, known for their firsthand experiences with cancer. These community partners (R.B., M.G., B.L., C.P.) underwent rigorous Community-Based Participatory Research training at Ponce Health Sciences University to ensure effective participant recruitment and engagement.

Inclusion criteria for participants encompassed individuals of any sex, aged 21 years or older, comprising healthcare providers (e.g., a practice of clinical psychology, social workers, physicians, and nurses of more than 20 patients coping with cancer), community partners (e.g., any community leader from the south area of Puerto Rico), cancer patients (e.g., a patient with active cancer diagnosis, OR a patient with cancer recurrence), and caregivers (e.g., any participant reported by the patient as their caregiver), including diverse community partners aimed to understand communication needs within the targeted community context comprehensively. Participants were selected based on referrals from our community partners and availability. Focus groups were used as they allowed us to investigate collective perspectives, attitudes, behaviors, and experiences, facilitating the acquisition of rich, in-depth data and uncovering both consensus and disparities within and between groups [20]. We conducted three focus groups and balanced the representation based on the community role of each group. We invited healthcare providers (*n* = 8), community partners (*n* = 8), patients (*n* = 8), and caregivers (*n* = 8), and the focus groups lasted for 60 to 80 min. On the day of the focal group, we ended up with patients (*n* = 8), caregivers (*n* = 8), community partners (*n* = 7), and providers (*n* = 4). We divided each of the three focus groups as follows: Group 1: patients (*n* = 3), caregivers (*n* = 3), community partners (*n* = 3), and healthcare providers (*n* = 1); Group 2: patients (*n* = 3), caregivers (*n* = 3), community partners (*n* = 2), and healthcare providers (*n* = 2); and Group 3: patients (*n* = 2), caregivers (*n* = 2), community partners (*n* = 2), and healthcare providers (*n* = 1). 

To adapt the communication intervention and follow the CER framework, the qualitative thematic content analysis of focus group transcripts, utilizing Nvivo v12 (2020), was employed. A semi-structured interview guide was used for the focus group and to guide the qualitative coding process, with a team of qualitative analysts (L.R.-R., T.R., Z.V.) independently coding transcripts. Constant discussions among analysts ensured consensus on coding interpretations, enhancing the rigor and reliability of the analysis. Continuous discussions established a comprehensive coding dictionary, facilitating consistent coding across all transcripts. 

### 2.1. Semi-Structured Interview

The focus group was guided through a semi-structured interview that covered questions regarding the development of a communication intervention. In addition, the discussions revolve around assertive communication training, examining its potential advantages, essential components, and perceived acceptability among patients and caregivers. In addition, the interview examined participants’ opinions on strategies for training effective communication skills, based on their experiences and knowledge. The study examined potential challenges in implementing communication skill training in various settings, including oncology clinics, support groups, and virtual platforms. Finally, the study also explored factors that could promote participation in the training within these settings.

### 2.2. Focus Group Interview

1. Thoughts on Communication Intervention:
  ○ What are your thoughts on a communication intervention for cancer patients and caregivers?
  ○ What do you think communication interventions typically involve?
  ○ What are your perceptions regarding the acceptability of a communication intervention?
2. Assertive Communication Training:
  ○ How do you think practicing expressing feelings, emotions, and decisions through role-playing exercises can be beneficial?
  ○ What aspects do you believe are important to include in these conversations?
  ○ How might this exercise benefit patient–caregiver communication?
  ○ How acceptable do you think assertive communication training would be for patients and caregivers?
3. Strategies for Communication Skill Training:
  ○ Based on your experience, what strategies do you think would be useful to include in communication skill training?
4. Anticipated Implementation Barriers:
  ○ What barriers to implementation do you anticipate in the following?
    - Oncology clinics;
    - Support groups;
    - Telephone intervention;
    - Virtual intervention;
    - Home visit intervention.
5. Potential Implementation Facilitators:
  ○ What factors do you think could facilitate participation in communication skill training in the following?
    - Oncology clinics;
    - Support groups;
    - Telephone intervention;
    - Virtual intervention;
    - Home visit intervention.

## 3. Results

The sociodemographic information of the participants indicates that 89% were female, while 11% were male. The average age among the participants was 53, and the average income was reported to be USD 25,880. Regarding marital status, 37% of the participants were married, 30% were single, 7% were widowed, and only 3% were divorced. Regarding participants’ characteristics, 30% of patients (*n* = 8) and caregivers (*n* = 8) represent each other, 26% were community partners (*n* = 7), and 15% were healthcare providers (*n* = 4). See Table 1. 

### 3.1. Acceptance of the Communication Intervention

When asked about their acceptance of a communication-based intervention, participants verbalized their acceptance of the intervention. Thus, patients (*n* = 6), community leaders (*n* = 7), healthcare providers (*n* = 4), and caregivers (*n* = 3) agreed to implement the intervention. Within the focus groups, patients reported, “…That intervention could be emotional or spiritual help…” (Participant #6). Furthermore, caregivers demonstrated acceptance of the intervention when reporting, “I believe that there should be an intervention in communication between the patient and the caregiver or those around him so that they can strengthen themselves” (Participant #8). On the other hand, healthcare providers provide acceptance for both patients and caregivers: “…I think it would be beneficial because, as she mentioned, both caregiver and patient are in a different world.” (Participant #1). Moreover, community partners verbalized their acceptance of the communication intervention regarding communication problems between patients and caregivers: “So if that person [the patient] does not trust the caregiver, communication between them [the patient and caregiver] will not occur.” (Participant #23). Table 2 shows several interactions regarding the acceptability of the communication intervention.

The participants accepted the Communication Skill Training intervention. When asked about the acceptance, patients reported, “…That intervention could be an emotional or spiritual help…” (Participant #6). Moreover, caregivers demonstrated acceptance of the intervention when reporting, “I believe that there should be an intervention in communication between the patient and the caregiver or those around him so that they can strengthen themselves” (Participant #8). Conversely, healthcare providers provide acceptance for both patients and caregivers: “I believe that emotional communication is part of, not only training the caregiver but also training the patient on how to communicate emotions assertively” (Participant #27). Within community leaders, they reported, “When you bring talks to communities of health professionals, people feel more confident and ask questions” (Participant #4).

### 3.2. Communication Strategies

According to the interactions with patients, caregivers, healthcare providers, and community leaders, they identified communication strategies that could be integrated into the intervention. When asked about the communication strategies, patients (*n* = 13), community leaders (*n* = 10), caregivers (*n* = 8), and healthcare providers (*n* = 5) reported the need to incorporate communication strategies to improve emotional support. Moreover, community leaders (*n* = 15), healthcare providers (*n* = 9), caregivers (*n* = 5), and patients (*n* = 6) expressed the importance of incorporating communication strategies to talk about cancer diagnosis. Another communication strategy is to improve communication between healthcare providers (*n* = 5), patients (*n* = 4), community leaders (*n* = 4), and caregivers (*n* = 1). Effective communication within the extended family can enhance support and understanding among patients (*n* = 4), healthcare providers (*n* = 3), and community leaders (*n* = 3). Finally, participants reported the need to talk about treatment and symptoms among community partners (*n* = 4), healthcare providers (*n* = 3), caregivers (*n* = 3), and patients (*n* = 2). See Table 3 for interactions and for illustrative quotes on strategies.

While discussing the theme of communication strategies, participants expressed the need for specific topics. As a part of the topic, emotional support arose as a benefit, while the patient reported, “The intervention could be an emotional help” (Participant #6). Additionally, community partners agreed with the need for emotional support for caregivers: “In addition to preparing…the ones who take care should be emotionally and psychologically prepared because they can be drained… These persons must be psychologically prepared to work with the patient” (Participant #23). In addition, to train about communication strategies, the patient reported that an educational component must be included: “If within the group of professionals, there was someone who could educate you from the beginning to be able to manage the impact it has on your life and the lives of those closest to you” (Participant #25). Furthermore, both caregivers report the beneficial impact of receiving a communication skill intervention: “Intervention in communication between the patient and the caregiver or those around them to empower them” (Participant #8), and healthcare providers report the need for a communication skill intervention: “We have had to work on how to express more assertively and affectionately” (Participant #27). 

### 3.3. Facilitators

When asked to discuss their views on the possible facilitators of the communication intervention development and implementation, community leaders (*n* = 11), patients (*n* = 8), caregivers (*n* = 6), and healthcare providers (*n* = 5) indicate how support groups play an integral role in the journey of cancer dyads and provide a supportive role during the process. Patients (*n* = 6) and healthcare providers (*n* = 6) also express the integration of healthcare providers in facilitators to the referral process of the intervention. Furthermore, community-based intervention was described as a facilitator by community leaders (*n* = 6) and healthcare providers (*n* = 6). Participants reported the facilitator on an institutional level by community partners (*n* = 6) and patients (*n* = 4). Phone intervention was presented as a facilitator by healthcare providers (*n* = 3), community partners (*n* = 2), and patients (*n* = 1). Virtual intervention was presented as a facilitator by community partners (*n* = 1) and caregivers (*n* = 1). For facilitators, see Table 4.

Through the discussion of facilitators of the CST intervention, caregivers reported that support groups would be facilitators because of the interaction between patients in those groups: “Many patients become counselors for other patients” (Participant #8). Moreover, community partners reported about the importance of the support groups: “So, support groups have a different role because through self-help [talks, workshops] to the partners, organization…” (Participant #26). Similarly, healthcare providers mentioned as facilitators that the CST intervention would be focused on the community, “Perhaps focusing more on the community as preparedness or prevention” (Participant #27). Participants describe the inclusion of providers as facilitators to the referral of the intervention: “Providers could refer patients to support groups… to help patients and caregivers” (P08). On the Institutional level, “organizations, for example, oncology clinics, could facilitate patients and space” (Participant #27). Finally, patients reported that home visits would be a facilitator due to the familiarity of the patient and caregiver with the environment: “You are in their environment” (Participant #6). 

### 3.4. Barriers

Participants also identified key barriers that could hinder or interfere with the intervention’s implementation. Patients (*n* = 6), community partners (*n* = 5), and caregivers (*n* = 3) reported barriers to non-financial assistance to support groups. Community leaders (*n* = 4), healthcare providers (*n* = 2), caregivers (*n* = 1), and patients (*n* = 1) recognize participants’ lack of transportation as a barrier. Patients (*n* = 4) and community leaders (*n* = 3) identified needing more support groups in each county. Moreover, participants reported a need for more skills in technology as barriers: caregivers (*n* = 4), community partners (*n* = 2), healthcare providers (*n* = 1), and patients (*n* = 1). The oncology clinic setting and administration, were referred to as a barrier by patients (*n* = 2) and community partners (*n* = 1). See Table 5 for intervention barriers.

When asked about the possible barriers in implementing the CST intervention regarding transportation, support groups, where the intervention takes place, etc., participants identified some challenges that may impede the implementation of the intervention. Patients reported non-financial assistance to support groups as a barrier because of schedule problems between the support groups and dyads; “But it is also the fact that the person has the accessibility to go [to activities, workshops]… because sometimes patients or caregivers are working and if the activity is in a conflicting schedule, they [patients and caregivers] will not be able to go” (Participant #25). Conversely, the lack of transportation is a barrier in implementing the intervention. Regarding the lack of transportation, healthcare providers reported, “Patients cannot go out of the house because of many difficulties, such as transportation” (Participant #27). In the same way, community partners identified the lack of support groups as a barrier by mentioning, “Not all places have support groups (Participant #3). Additionally, caregivers reported that implementing the intervention through the phone would be a barrier because “well, in terms of technology, it could be the signal, the technology by itself” (Participant #18).

## 4. Discussion

When participants (e.g., caregivers, patients, community partners, and providers) were asked about a communication intervention, all participants favorably accepted the need to adapt and implement a communication intervention for patients and caregivers coping with cancer. Specifically, participants reported the need to include strategies for psychological and emotional sharing, cancer diagnosis education, learning what to say, and communication with providers and extended family members. These findings are like other patient and caregiver research, where patients and caregivers expressed the acceptability of including communication skills strategies (e.g., psychological and emotional sharing, cancer diagnosis education, learning what to say, and improving general communication) in patients and caregivers’ care [21]. 

Moreover, participants reported facilitators for a communication skill intervention that includes implementation through support groups, healthcare providers, and community-based interventions. Similarly, the literature has established the benefits of providing support for patients and caregivers through community healthcare workers or support groups [8]. Research specifically shows that effective communication interventions encompass various elements such as problem-solving skills, mutual support, companionship, and acquiring strategies for discussing cancer diagnosis [16]. Notably, our study suggested some barriers that must be addressed, such as non-financial assistance to support groups, transportation, and a lack of support groups. Similarly, studies that focus on implementing an intervention for patients coping with cancer agree that transportation seems to be a challenge that impedes the implementation of an intervention [22]. Also, findings suggested some barriers in implementing the intervention through phone and virtual modalities. In this way, the literature emphasizes that, to implement an intervention, it is essential to address limitations regarding the barriers to internet access in order to ensure the intervention’s effectiveness [22].

These findings suggest the importance of integrating a communication intervention among our cancer community support groups. It emphasizes the need to include community partners in this psychosocial intervention’s development and implementation process. Th existing literature also adds information on how to use community members when implementing psychosocial interventions; aligning with findings from the existing literature, participants highlight the utilization of community members in psychosocial interventions [8]. Community partners are trusted figures who foster rapport and credibility, bolstering engagement and adherence to psychosocial interventions [10]. These findings highlight the importance of integrating psychosocial services within established community-based resources, such as support groups. Future studies will benefit from training community partners such as community healthcare workers to implement a communication skill intervention within the community, aiming to improve patient and caregiver communication skills.

## 5. Conclusions

There are specific communication needs faced by Latino patients and caregivers, and the present findings highlight the significance of tailoring communication skill interventions among participants coping with cancer [2]. Similarly, the literature’s recognition of the lack of adaptation for this demographic reinforces the importance of culture and the development of culturally tailored interventions focusing on practical communication skills [1]. Overall, the findings of this study highlight the acceptance and perceived benefits of a communication skill intervention among caregivers, patients, community partners, and healthcare providers. Through support groups, participants rated various communication strategies favorably and reported positive outcomes for caregivers and patients. The study aligns with the existing research, emphasizing the acceptability and importance of incorporating communication skill strategies into interventions for patients and caregivers [2]. It acknowledges the efficacy of interventions involving partners or support groups, particularly in improving communication among cancer patient–caregiver dyads. This study supports the need to integrate communication interventions into cancer community support groups and involve community partners in the implementation process. Utilizing community members, particularly those trained as community healthcare workers, can enhance engagement and adherence to psychosocial interventions. 

## 6. Limitations

Although the current study sheds light on the acceptability and facilitators of a communication intervention among cancer-coping participants, it is important to acknowledge several limitations. Firstly, the study’s sample size might limit the generalizability of the findings, as it may not adequately represent the diverse range of experiences and perspectives of cancer patients, caregivers, providers, and community partners. The team did not include a pilot test of the intervention, which was an additional limitation. We also did not have sexual orientation or sexual identity in the demographic data. By not collecting this critical information, we could not divide the responses by gender or sexual identity or consider the gender of the interviewer for the results of this study.

## Figures and Tables

**Table 1:** Sociodemographic characteristics.

Variables	f (%), $\bar{x}$
Participants’ Socio-demographic Characteristics	
Sex	27
Female	24 (89%)
Male	3 (11%)
Age	53
Income	25,880
Marital Status	
Married	10 (37%)
Single	8 (30%)
Widow	2 (7%)
Divorced	1 (3%)
Participant’s Characteristics	
Patients	8 (30%)
Caregivers	8 (30%)
Community Partners	7 (26%)
Healthcare Provider	4 (15%)

**Table 2:** Acceptability of communication intervention.

Topic	Interactions	P	C	HP	CP
Acceptability of Communication Intervention	20	6	3	4	7

Note: In this table, “P” represents patient, “C” represents caregiver, “HP” represents healthcare provider, and “CP” represents community partner.

**Table 3:** Communication strategy themes.

Topic	Interactions	P	C	HP	CP
Could improve emotional support	36	13	8	5	10
Cancer diagnosis education	35	6	5	9	15
Learn what to say	32	8	5	9	10
Improve general communication	19	3	4	8	4
Coping strategies	8	4	3	1	—
Communication with healthcare provider	14	4	1	5	4
Communication with extended family members	11	4	1	3	3
Treatment and symptom	12	2	3	3	4

Note: In this table, “P” represents patient, “C” represents caregiver, “HP” represents healthcare provider, and “CP” represents community partner.

**Table 4:** Facilitators.

Topic: Intervention Facilitators	Interactions	P	C	HP	CP
Support groups	30	8	6	5	11
Healthcare providers’ referral to the intervention	15	6	2	6	1
Community-based intervention	16	2	4	4	6
Home visit	11	2	3	3	3
Institutional level	10	4	—	—	6
Phone intervention	6	1	—	3	2
Virtual intervention	2	—	1	—	1

Note: In this table, “P” represents patient, “C” represents caregiver, “HP” represents healthcare provider, and “CP” represents community partner.

**Table 5:** Barriers.

Topic: Barriers	Interactions	P	C	HP	CP
Non-Financial assistance to support groups	14	6	3	—	5
Participants’ lack of transportation	8	1	1	2	4
Lack of support groups	7	4	—	—	3
Lack of technology skills	8	1	4	1	2
Home-based visits	3	—	—	—	3
Oncology clinic	3	2	—	—	1

Note: In this table, “P” represents patient, “C” represents caregiver, “HP” represents healthcare providers, and “CP” represents community partners.

## Data Availability

Data can be shared upon request.
